# 

**Published:** 1901-04

**Authors:** 


### BOOKS RECEIVED.

Fischer—Infant-feeding in Health and Disease. A modern book on all Methods of Feeding. For Students, Practitioners and Nurses. By Louis Fischer, M D , Attending Physician to the Children's Service of the New York German Poliklinik; Bacteriologist to St. Mark's Hospital; Professor of Diseases of Children in the New York School of Clinical Medicine; Attending Physician to the Children's Department of the West Side German Dispensary. Containing 52 illustrations, with 16 charts and tables, mostly original. 368 pages. Philadelphia: F. A. Davis Company. 1901. [Price, $1.50, net.]

Diseases of the Nose and Throat. By D. Braden Kyle, M. D., Clinical Professor of Laryngology and Rhinology, Jefferson Medical College, Philadelphia; Consulting Laryngologist, Rhinologist and Otologist, St. Agnes' Hospital. Second edition, revised. Octavo, 646 pages. Over 150 illustrations and 6 lithographic plates. Philadelphia and London; W. B. Saunders & Co. 1901. [Cloth, $4.00. net.]

The Treatment of Fractures. By Charles L. Scudder, M. D., Assistant in Clinical and Operative Surgery, Harvard Medical School. Second edition, revised and enlarged. Octavo, 433 pages, with nearly 600 original illustrations. Philadelphia and London: W. B. Saunders & Co. 1901. [Polished Buckram, $4.50, net.]

Progressive Medicine. Volume V., March, 1901. A Quarterly Digest of Advances, Discoveries and Improvements in the Medical and Surgical Sciences. Edited by Hobart Amory Hare, M. D., Professor of Therapeutics and Materia Medica in the Jefferson Medical College in Philadelphia, assisted by H. R. M.

Landis, M. D., Assistant Physician to the Out-Patient Medical Department of the Jefferson Medical College Hospital. Octavo, 440 pages. Illustrated. Philadelphia and New York: Lea Brothers & Co. [Per annum, in four cloth-bound volumes, $10.00.]

Cancer of the Uterus. Its Pathology, Symptomatology, Diagnosis and Treatment. Also the Pathology of Diseases of the Endometrium. By Thomas Stephen Cullen, M. B. (Toronto), Associate Professor of Gynecology in the Johns Hopkins University. Imperial octavo, pp. 709, with 11 lithographic plates and over 300 colored and black illustrations in the text, by Max Brodel and Hermann Becker. New York: D. Appleton & Co. 1901.

Manual of the Diseases of Children. By John Madison Taylor, A. M., M. D., Professor of Diseases of Children, Philadelphia Polyclinic; Assistant Physician to the Children's Hospital and to the Orthopedic Hospital; Neurologist to the Howard Hospital, etc.; and William H. Wells, M. D., Adjunct Professor of Obstetrics and Diseases of Infancy, Philadelphia Polyclinic; Instructor in Obstetrics in the Jefferson Medical College. Octavo, pp. 859. Illustrated. Second edition, thoroughly revised and enlarged. Philadelphia: P. Blakiston's Son & Co. 1901 [Price, $4.50.]

A Treatise on Appendicitis. By George Ryerson Fowler, M. D., Professor of Surgery in the New York Polyclinic; Examiner in Surgery, New York State Medical Examining Board; Surgeon to the Methodist Episcopal Hospital; Surgeon-in-Chief to the Brooklyn Hospital,etc. Octavo, pages, 235. Illustrated. Second edition, revised and enlarged. Philadelphia and London: J. B. Lippincott Company. 1900. [Price, $2.50.]

Pulmonary Consumption, Pneumonia and Allied Diseases of the Lungs. Their Etiology, Pathology and Treatment, with a chapter on Physical Diagnosis. By Thomas J. Mays, A. M., M. D., Professor of Diseases of the Chest in the Philadelphia Polyclinic; Visiting Physician to Rush Hospital for Consumption. Octavo, pages, 539. Illustrated. New York; E. B. Treat and Company. 1901. [Price, $3.00.]

Hypnotism. A Complete System of Method, Application and Use, prepared for the self-instruction of the Medical Profession. By L. W. De Laurence, Instructor at the School of Hypnotism and Suggestive Therapeutics, Pittsburg. Illustrated. Chicago: The Henneberry Co. 1901. [Price, $1.50.]

The History of Medicine in the United States. A collection of facts and documents relating to the history of medical science in this country, from the earliest English colonization to the year 1800. With a supplementary chapter on the discovery of anesthesia. By Francis Randolph Packard, M. D. Octavo, pp. 542. Illustrated. Philadelphia: J. B. Lippincott Co. 1901. [Price, $4.00.]

The Student's Manual of Venereal Diseases. By F. R. Sturgis, M. D., sometime Clinical Professor of Venereal Diseases of the Medical Department of the University of the City of New York, etc. 12mo, pp. 216. Seventh edition, revised and in part rewritten by the author and Follen Cabot, M. D., Instructor in Genito-urinary and Venereal Diseases in the Cornell University Medical College. Philadelphia: P. Blakiston's Son & Co. 1901. [Price, $1.25.]

Retinoscopy. In the determination of refraction at one meter distance, with the plane mirror. By James Thorington, A. M., M. D., author of "Refraction and How to Refract;" Professor of Diseases of the Eye in the Philadelphia Polyclinic and College for Graduates in Medicine. 12mo, pp. 89; 51 illustrations, 12 of which are colored. Fourth edition, revised and enlarged. Philadelphia: P. Blakiston's Son & Co. 1901. [Price, $1.00.]

Harrington's Practical Hygiene. For Students and Practitioners of Medicine and Medical Officers By Charles Harrington, M. D., Assistant Professor of Hygiene in Harvard Medical School, Boston. In one very handsome octavo volume of 718 pages, with 105 engravings and 12 full-page plates in colors and monochrone. Lea Brothers & Co., Publishers, Philadelphia and New York. 1901. [Cloth, $4.25, net ]

